# From Pregnancy to Postpartum: The Dynamic Reorganization of the Maternal Brain

**DOI:** 10.1177/26331055251315488

**Published:** 2025-01-19

**Authors:** Natalia Chechko, Susanne Nehls

**Affiliations:** 1 Department of Psychiatry, Psychotherapy and Psychosomatics, Faculty of Medicine, RWTH Aachen, Aachen, Germany; 2 Institute of Neuroscience and Medicine, JARA-Institute Brain Structure Function Relationship (INM 10), Research Center Jülich, Jülich, Germany; 3 Institute of Neuroscience and Medicine, Brain and Behavior (INM-7), Research Center Jülich, Jülich, Germany

**Keywords:** Postpartum, maternal brain, neuroplasticity, hormonal fluctuation, microglia

## Abstract

The postpartum period is marked by radical changes in the maternal brain. Seeking to explore the mechanisms that underlie these changes, this article focuses on the relevant hormonal, inflammatory, and behavioral factors. Longitudinal imaging studies have shed valuable light on both short- and long-term alterations in postpartum brain structure and connectivity, particularly in the regions that play key roles in emotion regulation and stress response. It is plausible that these peripartum changes contribute to the mental health challenges new mothers face, including postpartum depression. Adding to our understanding of postpartum neurobiology, this insight highlights the importance of personalized intervention in the promotion of maternal well-being.

## Brain Changes During Pregnancy

It has been found that steroid hormones exert a profound influence on brain function through mechanisms such as neurotransmitter system modulation and synaptic plasticity. Their impact is particularly dramatic during pregnancy. The level of increase in circulating steroid hormones over a 40-week gestational window remains unsurpassed in a women’s life.^1,2^ These continually increasing hormones are thought to change the pregnant brain, both in animals and humans, by crossing the blood–brain barrier.^2,3^

Prior research has shown that pregnancy leads to a reduction in gray matter volume (GMV), cortical thickness and white matter density.^4-8^ Animal studies suggest that these changes may be driven by hormonal influences on cellular morphology. The administration and withdrawal of progesterone and estrogen have been found to affect dendritic spine density along with dendritic length/branching/soma size alterations within the hippocampus or the medial preoptic area.^9-11^ The brain’s immune cells, microglia, are also sensitive to the gonadal hormones, showing reduced density during late pregnancy and the early postpartum period in areas such as the amygdala, the medial prefrontal cortex, the nucleus accumbens and the hippocampus.^12^

In addition, while some brain changes have been found to be restricted to pregnancy and the postpartum phase, others are long-lasting. For instance, irrespective of age, (multi)parity appears to have a lasting impact on the maternal brain, particularly on microglial activity and the cell signaling pathways.^13^

## Postpartum Brain Reorganization

Confirming the pregnancy-related decline, our previous research has shown widespread GMV decrease in the maternal brain, compared to nulliparous women, in regions including the hippocampus/amygdala, the cingulate cortex, the medial orbitofrontal cortex, the insula and the basal ganglia at 2 days after childbirth (the acute postpartum period).^14,15^

Our multimodal longitudinal imaging data, gathered through the Risk of Postpartum Depression (RIPOD) study,^15,16^ have indicated further regionally distinct short- and long-term effects on the reorganization of postpartum brain structure and function.

In healthy postpartum women, the hippocampus, the amygdala and the subgenual prefrontal cortex have been found to undergo significant alterations in volume and cortical thickness in the acute to subacute postpartum period (ie, until 6 weeks postpartum).^14,15^ The cortical thickness and GMV of the subgenual and lateral prefrontal cortices do not return to the pre-pregnancy levels even at 12 weeks postpartum.^15^ According to our preliminary data, changes continue in the cingulate cortex, the temporal and parietal cortices, the amygdala, the hippocampus, and the insula even at 6 months postpartum. The early postpartum period is also associated with pronounced alterations in resting-state connectivity.^17^

The interregional resting-state connectivity with the bilateral putamen was found to normalize to the relevant level of nulliparous brains within 6 to 9 weeks postpartum, while intraregional changes in the resting-state insula activity persisted throughout the entire follow-up period of 6 months.^17^ The reduced interregional resting-state connectivity showed a temporal association with blood progesterone concentration (the suppressed progesterone level was temporally associated with reduced interregional resting-state connectivity). Thus, our finding indicates that the most dynamic reorganization of the postpartum brain structure primarily occurs within the initial postpartum weeks, and involves regions responsible for emotion and stress regulation, highlighting a relationship with peripartum hormonal adaptation.

## Hormonal, Behavioral and Physiological Influence on Postpartum Neuroplasticity

Hormonal shifts may have the strongest influence on the temporal trajectories of maternal brain changes. Our data show that the gradually increasing postpartum blood progesterone levels closely correlate with the temporal patterns of postpartum GMV changes and resting-state connectivity.^15,17^ Regions with a high receptor gene expression are particularly involved during the early postpartum period, while areas with GABAa and glutamate receptor gene expression become more prominent from 12 weeks to 6 months postpartum (preliminary unpublished results). Additionally, the third-trimester urine estradiol levels appear to correspond to GMV changes from pre-pregnancy to 1-4 months postpartum, potentially contributing to the overall pattern of neural alterations.^5^

Besides the hormonal factors, interaction with the child appears to contribute significantly to the shaping of the postpartum brain. For instance, volumetric changes in the bilateral amygdala, the temporal pole, the right olfactory gyrus, the left anterior cingulate, the bilateral thalamus, and the cerebellum were found to predict less hostile behavior toward the child from the postpartum through the follow-up periods.^15^ These findings underscore the relevance of both biological and experience-based factors to maternal brain changes, highlighting the complex hormonal and behavioral regulation of postpartum brain plasticity. However, exactly how hormonal fluctuations influence the early-postpartum brain changes, and the likely impact of behavioral and psychological adaptations on long-term functional changes, remain to be determined. These dramatic and complex changes, involving hormonal, metabolic and behavioral factors, may contribute to the development of depressive disorders in some mothers, with possible long-term consequences with respect to mental and physiological health.

## Health-Related Factors for Postpartum Neuroplasticity

The neurobiological reorganization in the postpartum period may contribute to the adaptive and risk processes associated with motherhood. Our longitudinal RIPOD study has revealed that roughly 10 % of women experience PPD with the onset occurring within the first 6 postpartum weeks. Our data also demonstrate that 80% of women with PPD show early symptoms of “baby blues,” with premenstrual syndrome being identified as a risk factor for more severe postpartum mood disorders.^18^

In addition, neuroinflammatory and metabolic processes during pregnancy may contribute to brain structure changes, elevating cognitive and neurodegenerative disease risks in later life.^19,20^ Gestational diabetes and hypertensive disorders, for instance, have been found to be associated with postpartum mood disorders and cognitive impairments.^21,22^ Hippocampal GMV reductions are linked to inflammation-mediated effects in unipolar depression and schizophrenia.^23^

Moreover, sex and gender significantly influence both the vulnerability and resilience to mental disorders.^24^ While the adoption of SAGE (Sex and Gender Equity in Research) reporting guidelines has improved the inclusion of sex- and gender-specific analyses in scientific studies, more needs to be done with respect to the promotion and implementation of these guidelines. To address this, two ongoing projects in our working group at the psychiatric hospital at UK Aachen are investigating the short- and long-term effects of hormones on the maternal brain, as well as postpartum risk factors such as obesity, multiparity, diabetes, and pregnancy interruption.

In summary, the postpartum period involves a significant reorganization of the brain based on hormonal, inflammatory, and behavioral factors. While some changes are short-term, others tend to persist, affecting maternal health and adaptation to motherhood. Future research needs to shed more light on these mechanisms, and the relevant risk factors, in order that appropriate interventions may be made to meet the treatment needs of postpartum women.
